# Review of Extracting Information From the Social Web for Health Personalization

**DOI:** 10.2196/jmir.1432

**Published:** 2011-01-28

**Authors:** Luis Fernandez-Luque, Randi Karlsen, Jason Bonander

**Affiliations:** ^3^Division of Knowledge ManagementCenters for Disease Control and PreventionAtlanta, GAUnited States; ^2^Computer Science DepartmentUniversity of TromsøTromsøNorway; ^1^Northern Research InstituteTromsøNorway

**Keywords:** Medical informatics, Internet, information storage and retrieval, online systems, health communication, data mining, natural language processing

## Abstract

In recent years the Web has come into its own as a social platform where health consumers are actively creating and consuming Web content. Moreover, as the Web matures, consumers are gaining access to personalized applications adapted to their health needs and interests. The creation of personalized Web applications relies on extracted information about the users and the content to personalize. The Social Web itself provides many sources of information that can be used to extract information for personalization apart from traditional Web forms and questionnaires.

This paper provides a review of different approaches for extracting information from the Social Web for health personalization. We reviewed research literature across different fields addressing the disclosure of health information in the Social Web, techniques to extract that information, and examples of personalized health applications. In addition, the paper includes a discussion of technical and socioethical challenges related to the extraction of information for health personalization.

## Introduction

The use of the Web by health consumers and professionals has changed with the emergence of the Social Web. This phenomenon has been described as Medicine 2.0 [1]. Whereas 10 years ago the Web was coming into its own as an e-commerce engine, the last 5 years have seen an increase in social interaction and content creation platforms that further engage and enmesh individuals in each other’s online lives, increasing the sharing of knowledge. This is especially true and important for individuals seeking health information and interested in finding others with health conditions like their own. Health consumers are socializing, searching for health information [2,3], and creating content about their health in user profiles, blogs, or videos [4]. Sharing experiences and knowledge can go beyond traditional Web content and include structured health data in sites like PatientsLikeMe [5] and 23andMe [6].

The phenomenon of the Social Web would not have been possible without the transformation of Web content from static to dynamic thus providing a much richer interactive Web experience. With the emergence of the adaptive Internet in the early 1990s, websites started to change dynamically, making it possible to provide different Web content for each user. As early as 1994, the system MetaDoc changed the content of technical Web documentation based on level of expertise of the reader [7]. This adaptation of the content for a specific user is known as Web personalization [8] and adaptive hypermedia [9]. Web personalization is making the Web more efficient when accessing information and services. For example, when buying a book at Amazon.com, related recommendations are based on browsing history.

Personalization is also used to adapt Web health information and applications to the needs of each user. As explained in the background section, health education since the 1990s has been personalized and delivered through the Web with positive patient outcomes [10].

One of the main challenges when creating personalized health applications is to capture the information needed for personalization. Traditionally, information capture has relied on input from users (eg, questionnaires), which is time consuming and may undermine the interest of users. A new approach is emerging that consists of using the Web itself as a source of information for health personalization. For example, personal health records (PHRs) integrate many personalized applications, such as the online service TrialX that recommends clinical trials to health consumers based on their PHRs [11]. Content generated by health consumers can also be used for personalization. For example, in the project RiskBot, some methods have been developed for personalizing health information using data from users’ profiles in MySpace [12,13]. These are just some of many examples illustrating the different possibilities for extracting information from the Social Web for health personalization.

The objective of this paper is to provide a review of the different approaches for extracting information from the Social Web for health personalization. The paper is structured as follows: the background section provides an introduction to health personalization across different research areas using as an example the case of Tailored Health Education. In the following section, we review approaches to extract information for personalization from different sources of information available in the Social Web. In the discussion section, we address current and future challenges including both technical and socioethical issues. Finally, in the conclusion we summarize the main contributions of the paper.

## Methods

In this review, our search strategies were designed to identify relevant research literature that addressed the following aspects of health personalization in the Social Web: (1) studies about the disclosure of health information in the Social Web, (2) techniques to extract that information, and (3) examples of applications. Major scientific databases in computer science (eg, ACM Digital Library) and biomedicine (eg, PubMed) were searched. In addition, we searched through the references of the selected papers, contributions to conferences, and nonresearch literature (eg, websites, books, technical reports). The background section provides an overview of the different research areas where the search was performed.

The multidisciplinary team of authors performed the selection and analysis of the relevant articles. Their backgrounds cover the different domains of the review (eg, information retrieval, computer science, health informatics, and public health). The different studies were analyzed to understand the implications for health personalization, including technical and socioethical aspects.

## Background

### Personalization

Personalization is a popular term with different meanings across domains. While personalization is the adaptation of something to a certain individual, there is a wide range of things that might be personalized (eg, treatments, websites, educational brochures, advertisements). In addition, personalization can be based on many different characteristics (eg, age, name, and location).

In the Web domain, personalization is the selection and adaptation of websites according to user specific characteristics or behaviors [8]. This is in contrast to “customization” or “adaptable systems,” which refer to systems that are adapted by users themselves, for example, modifying search retrieval preferences or portal settings [9].

In medicine, the term personalization typically refers to delivering health care interventions that are designed for an individual patient (eg, drugs designed for patients with a certain genetic characteristic) [14]. However, the meaning of the word personalization varies within the health domain. In the field of tailored health education, personalization can be as simple as using the patient’s name in the educational material. In that domain, personalization is a subtype of tailoring. Computer tailoring in health education has been defined as “the adaptation of health education to one specific person through a largely computerized process” [15].

For the purposes of this paper, we will use the definition of Web personalization [8] applied to the health domain. Therefore, we define Web health personalization as the adaptation of health-related Web content and applications to characteristics associated with a specific user.

### Relevant Research Areas 

There are different areas of research within health informatics (see Table 1) dealing with aspects related to the acquisition of information from the Social Web for health personalization. Tailored health education, the next subsection, is of special interest because in that domain, personalized Web applications have been used for more than a decade. In addition, there are relevant research areas in computer science, which are listed in Table 2.

**Table 1:** Relevant research areas in health informatics

Research Area	Importance for Health Personalization
Tailored health education [10]	Personalization of educational Web content to promote health and modify health behaviors
Personal health records [16]	PHRs are a source of information about users. Personalized applications can be integrated as third party applications inside the PHRs.
Biomedical text mining	Data mining techniques to extract information from text, for example, automatic classification of forum posts [17]
Consumer health vocabulary [18]	Study of the vocabulary used by health consumers and how it maps with medical standardized vocabulary
Computer-aided diagnosis	Analysis of text, audio, and video for diagnosis, for example, speech analysis in neurology [19]

**Table 2:** Relevant research areas in computer science

Research Area	Importance for Health Personalization
User modeling and personalization	Adaptation of Web systems to users and user modeling [8]
Computer vision	Extraction of information from images and videos, for example, age-group classification from facial images [20]
Affective computing and social signaling	Extraction of information about users emotions [21] and social behavior [22]
Collaborative computing	Use of collaborative techniques to build personalized systems and classify content, for example, tagging of Web content [23]
Web data mining	Extracting information from the Web, for example, the analysis of the links to find relevant websites [24]

### Tailored Health Education

The origin of Web health personalization is found in the field of tailored health education. Computers have been used to personalize health education from the early 1990s, including Web educational content. Detailed reviews of personalized health education can be found in Vries et al [15], Cawsey et al [25], and Kukafka et al [26]. Reviews dealing with Web-based interventions can be found in Lustria [10], Webb et al [27], and in Enwald et al about obesity [28].

**Figure 1:**
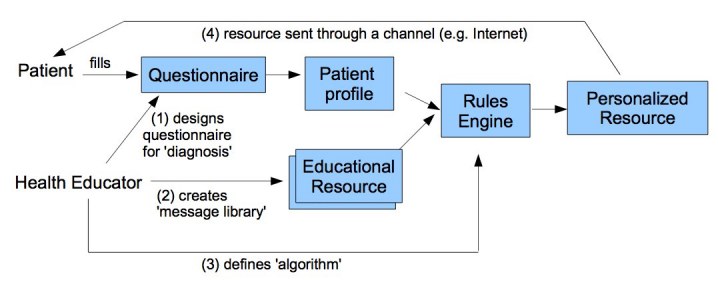
The process of tailoring health education

According to de Vries and Brug [15], the process of personalizing educational materials (see Figure 1) requires: 

at least: (1) a “diagnosis” at the individual level of characteristics that are relevant for a person’s health behavior or illness; (2) a “message library” that contains all health education messages that may be needed; (3) an “algorithm,” a set of decision rules that evaluates the diagnosis and selects and generates messages tailored to the specific needs of the individual user; and (4) a “channel.”

Using computer science terminology, the *diagnosis* can be seen as *user modeling* and the *message library* could be seen as the repository with the Web content to personalize. Different adaptations are possible within personalized health education such as selecting which content is to be presented, ordering of content, and adaptation of content itself.

As described in Figure 1, most personalized health education systems can be seen as expert systems where the expertise of a human health educator is captured to create a personalized intervention (eg, text message or website) based on a set of parameters about the patients and the educational resources. The parameters can be diverse, from basic demographics to complex psychological parameters, depending on the goals of the application. For example, physiological parameters may be more relevant to modify behaviors (eg, smoking) than to provide health information to patients with cancer. In most cases, the parameters about patients are captured using questionnaires, which are time consuming and may decrease the interest to participate. To alleviate that problem, certain parameters (eg, demographics and diagnosis) can be captured from electronic medical records [29].

Adapting the content itself can simply mean adding the patient’s name in the appropriate places. It can also mean to adapt content based on behavioral parameters grounded in models such as the transtheoretical model of health behavior change [30]. Personalizing an educational brochure about smoking cessation, for example, may only add the name of the smoker to the educational materials. A more complex personalization will be to provide content with different tips depending on whether the smoker is simply contemplating quitting or has decided to quit but is worried about “side effects” (eg, gaining weight). The adaptation can also be based on demographic information such as age and gender; for example, teenagers may consider quitting smoking mainly because it damages their image (eg, yellowing teeth) and not so much because it increases the risk of cancer. 

## Extracting Information From the Health Social Web

To create personalized health applications, it is necessary to acquire information about users. The information can be as simple as general demographic data (eg, age, gender, ethnicity, and location) or more complex, such as data acquired through structured questionnaires, health records, and so on. It is equally important to have adequate information about the Web content itself such as topic, language style, and date.

As summarized in Table 3, there are many information sources in the Social Web that can be used to extract information about users and content. The Social Web has facilitated the creation of Web content (eg, blogs, videos, and user profiles). User-generated content can be analyzed to extract information about Web content or users. In addition, user-generated content has been found to contain disclosed personal health information [31,32]. Further, many other sources of information are available such as ratings, links, and Web usage data (eg, click history). Finally, while not necessarily a part of the Social Web per se, personal health records (PHRs), if shared, represent a rich source of health information from which applications and services could be personalized. In the following subsections, we provide a description of different approaches to extract relevant information for health personalization from sources in the Social Web.

**Table 3:** Main sources of information for health personalization in the Social Web

Sources	Examples of Information That Can Be Extracted for Health Personalization
Personal health records[16]	Personal health information (eg, diagnoses and treatment) Demographic information Genetic information (eg, rare mutations) [33]
Textual content	Textual content is present in most of the Web content, and it can contain information about the authors or about the content itself (eg, description of a video).
User profiles in online communities	Health risk behaviors (eg, smoking) Demographic information [12,13,31] User preferences (eg, topics of interest) [34]
Forum posts and comments	Personal health information (eg, diagnoses and treatments) [32] Emotional/mental status of users [35] Type of content (eg, informational or conversational) [36]
Search queries	User interests [37]
Tags	Topics of tagged content and users interests [38]
Audio	Users emotional status [39,40] Diagnosis (eg, depression) [41]
Facial photos	Emotions [42], gender [43], and age [20]
Videos	Diagnosis (eg, neurological diseases) [44] Characteristics of videos (eg, topic and style) [45]
Ratings	Users preferences and similarities [46]
Social networks and links	Community discovery [47,48] Characteristics of Web content [24,49]
Web usage data	Classification of users based on navigation patterns (eg, clicks and browsing data) [50]

### Personal Health Records

Personal health records (PHRs) are lifelong electronic sources of personal health information controlled and managed by health consumers to support decision making [16,51]. The information contained within PHRs is generated by both clinical encounters and patients themselves. Web-based PHRs are becoming increasingly available in the United States [52].

The information contained within a PHR can range from general demographics to clinical visit information, lab test results, and genetic information [16,33]. Many currently available PHRs are beginning to comply with emerging data and interoperability standards like those found with the continuity of care record (CCR), clinical document architecture (CDA) and Health Level 7’s (HL7’s) PHR functional model. These not only facilitate interoperability with electronic medical records (EMR) but also provide a foundation from which health applications and services can be developed.

As PHRs begin to integrate with third party applications, a larger application ecosystem is fostered, which layers additional functionality provided by the third party applications [53,54]. That approach is similar to the iTunes App Store. For example, in Microsoft HealthVault alone, there are currently upwards of 50 different third party applications [55], a good example of which is TrialX [11]. TrialX uses the data from the PHRs to find possible subjects matching the inclusion criteria in clinical trials. In the PHR Indivo, a clinical trial evaluated the use of PHRs for delivering influenza prevention education [56].

Apart from PHRs, there are patient social networking sites offering users the option to share and visualize detailed and structured personal health information within a community, for instance PatientsLikeMe [5]. However, they have yet to provide application programming interfaces (APIs) for the integration of third party applications. Some researchers are looking into the integration of PHRs with social networking [54,57].

### Textual Content

Unstructured free text is one of the most common types of generated content in the Web. As explained in the following subsections, that textual content can be from different sources: (1) user profiles, (2) forums, blogs, and comments, (3) search queries, and (4) tags.

The use of natural language processing (NLP) is the most common approach to extract information from free text. NLP is defined as the use of computer algorithms to process written and spoken human language [58]. Processing text using NLP involves several phases. It includes the extraction of keywords, stop-word removal (eg, removal of irrelevant words), word sense disambiguation and stemming (reduce words to its root). With the extracted terms, different techniques can be used to analyze them, such as terms weighting, semantic networks, and advanced data mining techniques. NLP techniques to analyze text have been enhanced with semantic technologies so that domain knowledge is taken into account in order to alleviate the ambiguity of the extracted terms [59].

Despite the scarce examples where NLP has been used to analyze health content in the Internet, it has been widely used in the biomedical domain. For instance, NLP is used to analyze biomedical text and to create information retrieval applications [60]. As a result of many years of research, several open source frameworks have been developed, such as the Unified Medical Language System (UMLS) Knowledge Source Server [61,62]. This framework provides NLP tools for analyzing biomedical text and semantic networks for matching extracted terms with standardized vocabularies.

The application of biomedical NLP for the analysis of text generated by health consumers is challenged by the gap between the medical vocabulary and the vocabulary used by the health consumers. For example, the common expression “kidney stones” may refer to the medical term *kidney calculi*. It has been found that between 20% and 50% of health consumers’ expressions were not represented by professional health vocabularies [18,63]. Nevertheless, these studies imply that nearly half of the free text created by health consumers can be mapped directly to standardized medical vocabularies. Similar results have been found in self-reported symptoms of patients in PatientsLikeMe.com [64] and search queries in the MedlinePlus health portal [65]. In addition, an approach for the identification of new terms has been developed to create a consumer health vocabulary [66]. It consists of the use of NLP to find relevant terms and map them to standardized medical vocabularies. Then, the unmapped terms are classified manually and added to the consumer health vocabulary. Another possible approach to overcome the gap between the vocabularies is to recommend standardized medical terms while typing [67].

#### User Profiles in Online Communities

Users in social networks and online communities maintain a personal Web site with information about them. Many of these user profiles contain personal information, such as age, gender, and hobbies. Also a significant number of users disclose health information in these profiles. For example, a study found that the majority of the teenagers in MySpace are not just disclosing general demographic information but also information about their health risk behaviors (eg, alcohol abuse) [31]. In health social networks, such as TuDiabetes.com, many users disclose personal health information (eg, type of diabetes or latest blood glucose levels). A special case is PatientsLikeMe [5] where users disclose detailed health information in their profiles.

The automatic extraction of health information from profiles in social networks has been studied in the RiskBot project. In that project, NLP techniques were used to crawl, that is, explore, sex-seeking websites and classify behaviors exhibited on those sites into different risk categories with the intent of using this information to create personalized public health messages [12,13]. The same technique was recently used to extract obesity and its comorbidities from text-based hospital discharge summaries [68].

Outside the health domain, user profiles have been used to extract information about users’ interests to provide recommendations and to find users with similar interests [34].

#### Forum Posts, Blogs and Comments

In addition to user profiles, health consumers are generating significant amounts of textual content through blogs, posts in forums, microblogs, and comments. This content ranges from deeply personal narratives to recommendations and reviews to discrete pieces of health data. Several studies have found disclosed personal health information in different types of content (eg, Twitter [69,70] and YouTube [32]). For example, a simple search in Twitter for “#bgnow” returns tweets that include blood glucose levels. In the studies about Twitter, the extracted information was not used for personalization but was used to study the misuse of antibiotics [69] and to analyze and track sentiments, attitudes, and behavior during a pandemic [70].

Information extracted from content can also be used to gather more information about the content itself. For example, NLP techniques have been used to classify topics of health forums [17]. In this example, the posts in a medical forum were analyzed to extract terms from a predefined set of terms. Then, different data mining techniques were used to categorize the posts.

Web content can also be classified according to emotional parameters, such as intentionality, relying on the fact that the human language provides clues about emotions and intentions. The capture of these clues is being addressed in different research fields, such as affective computing [21] and opinion mining [71]. For example, a blog post can be objective and informative (eg, how to take an insulin injection) or be affective and raising a debate (eg, hate insulin injections). Techniques have already been developed outside the health domain to automatically classify posts depending on their informative nature [36]. In the health domain, similar techniques have been used to classify suicide notes [35] and preliminary work has been done in online suicide notes [72].

#### Search Queries

Search engines are among the most popular tools to search health information [3]. Many search engines store the text entered by the users to model the previous search queries and personalize the results.

In the health domain, there are only a few examples of health search engines using search queries for personalization. These techniques are mainly used in search engines of research literature [73]. In the health portal MedlinePlus, search queries have been used to analyze the vocabulary of the health consumers [65]. However, that information is used to detect misspellings and topics of interest and not to personalize the search results.

#### Tags

Nearly one third of Internet users in the United States have already tagged content [23] and 6% of the health information seekers have tagged or categorized Web health information [4]. Prior to the Social Web, many indexing techniques were based on taxonomies created by experts. Today, users are indexing content with their own tags that can be used collaboratively by utilizing new taxonomies of Web resources, known as “folksonomies”. In addition to classifying Web content, tagging is also used to capture information about the users. For example, the tagging history of users can be used to model their interests [38].

Health-related examples of tagging are found in platforms such as TuDiabetes.com [74] and GetHealthyHarlem [75], where tags are used to search and recommend content. One of the challenges with tagging is the appearance of ambiguity between tags. The integration of tags with ontologies opens many opportunities for using semantic-enhanced techniques [76], such as giving recommendations of tags based on medical ontologies [67]. It has also been found that nearly half of the tags created by patients for describing symptoms were found in medical standardized vocabularies [64].

### Images, Video, and Audio

In the Social Web, users are creating a wide variety of content apart from the text. Video, images, and audio are gaining in popularity as vehicles for sharing experiences and opinions. Extracting information from these file types, while of interest for personalization, has its challenges. The challenges result primarily from increased interpretive ambiguity in visual and audio processing and the computational cost. While the authors are not aware of explicit projects focused on extracting information from video within the Health Social Web, there are examples in other areas of research for instance computer vision, social signaling, affective computing, and computer-aided diagnostics.

Computer vision is concerned with computer systems that extract information from images. Computer vision techniques are used in many different domains (eg, computer-aided diagnostics). There are many examples of applications that extract information from people’s facial photos about emotions [42], gender [43], and age group [20].

In social signaling [22], behavioral cues (eg, vocal behavior and hand expressions) are extracted from audio, video, and pictures in order to produce a “social signal” with the meaning of the extracted information. For example, through analyzing the speech in a dialog it is possible to gather information about the emotional status of the speakers and their different roles [39,40].  

Social signaling is related to affective computing [21], which aims to create systems and devices that are adapted to human emotions. Affective systems have to recognize emotional information such as the “happiness” of a video [45] or the emotional expressions in a facial photo [42].

Computer-aided diagnostics use video and audio analysis to help diagnose different pathologies. For example, voice has been used to reveal patterns in the voice of patients with depression [41] and speech alterations in neurological disorders [19]. Video has been used to quantify the tremor in patients with Parkinson [44].

### Ratings

The ability to rate content is one of the most common types of feedback in the Social Web. It is used in a wide variety of collaborative filtering applications such as recommender systems [46]. The objective of these applications is to provide personalized recommendations based on what the system knows about “you” in conjunction with what it knows about “people like you”. As explained in Schafer et al [46], there are two main approaches to giving recommendations based on ratings: item-based and user-based. Item-based recommender systems will recommend highly rated items similar to those the specific user liked before. In the case of user-based systems, the rating history of a specific user will be used to find users with similar interests. The items with highest ratings among these like-minded users will be recommended. The rationale behind item-based systems is that “people who like x also like y,” while the rationale behind user-based systems is that “people similar to you also like y.”

Some applications are based on ratings in the health domain. For example, the health portal HealthyHarlem integrated a rating-based recommender system of health information [77]. There are also websites with ratings of health-care providers both in the United Kingdom [78] and the United States [79]. Integration of end-user and professional ratings has been explored in the project MedCertain [80] for creating a collaborative health information filtering system.

### Social Networks and Links

In many cases, the terms “online communities” and “social networks” are used indistinguishably. However, an online community is a subtype of social network where different users interact virtually, normally sharing specific goals. A social network, in the general sense, can be any network between people, such as family networks. The study of social networks predates the Web, and it has been used in health research [81]. As explained below, social network analysis has influenced how we browse and search the Web.

Similar to human social networks, the Web is a complex network of nodes (eg, websites) that are interconnected using links. The analysis of the “linking” structure among the different websites is a common source of information about websites [24]. A link is an implicit source of information about the “authority” or “prestige” of a website. For example, an outgoing link often indicates conveyance of authority to the linked website. That principle is the basis of many Web search algorithms, such as Google’s PageRank [82].

Link analysis algorithms originated from social network analysis (SNA). SNA has been used for decades as a tool to understand complex human social networks. For example, using SNA and longitudinal data from a population of people over a period of 30 years, Christakis and Fowler found important relationships between health behaviors and health risk as a product of the structure of social networks [81]. SNA has acquired more attention for the analysis of Web social networks since the Internet has become a major social platform where millions of users are establishing relationships of diverse types (eg, friends, fans, and followers).

In the domain of the Health Social Web, SNA has been used to study online communities [83]. In other Web domains, SNA has been used to extract information for personalization. For example, SNA has been used to infer characteristics (eg, centrality, reputation, and prestige) of the members of a community (eg, bloggers) [84]. That information can be used to identify nontrusted users who are more likely to have low quality ratings and content [85,86]. Another feature of SNA is the possibility to detect communities within large social networks [47,48]. The information about the subcommunities can be used for personalization. For instance, a blog about cancer from the community of forensic pathologists may not be the best to recommend to a health consumer.

Furthermore, a social network can be itself a personalization engine where users are spreading content through their friends. Individuals are using information about their friends to spread the Web content in a manual-personalized manner. This new “viral” pattern of distribution of Web content is being used in public health [87-89]. For example, the New York City Department of Health and Mental Hygiene designed an application in Facebook that let users send “e-condoms” as a mean of promoting safe sex for HIV prevention [89]. The analysis of the structure of the social network can be used to increase the dissemination of the information in viral applications by identifying users with higher influence [90].

### Web Usage Data

The extraction of Web usage data for Web personalization predates the Social Web, yet it is still widely applied. Web servers store information about users accessing websites, such as version of the Web browser, IP addresses, and clicked links. That information can be used to improve the design of a website (eg, making the most clicked elements more visible) and to personalize the interface (eg, personalizing the layout of the Web based on the size of the screen). Mobasher [50] reviews the wide range of techniques available to extract Web usage data for personalization.

Web usage data is collected in many health-related websites, such as in WebMD [91] and MedlinePlus [92]. In WebMD, Web usage data is used for personalizing the advertisements based on the type of user’s Web browser. Web usage data has also been used to evaluate the impact of public health interventions [93].

## Technical and Socioethical Challenges

As explained in the previous section, there are many possible approaches to extracting information for health personalization for the Social Web. However, these approaches have different implications, and how to apply them in personalization will vary depending on the context of the application. In order to decide which approach is the most suitable for a specific application, it is necessary to take into account the main technical and socioethical challenges arising from applying these approaches in health personalization. These challenges are addressed in the following subsections.

### Technical Challenges

There is a set of technical challenges associated with the approaches addressed in the previous sections. While it is not feasible to cover all the challenges with each approach, the discussion will focus on what we consider to be the most important ones related to health personalization (Table 4).

**Table 4:** Main technical challenges of extracting information from the Health Social Web

Challenges	Description
Relevance [94]	To determine which information is relevant for personalization is complex, and it depends on the objectives of the personalization.
Reliability and validity	The reliability and validity of the information used for personalizing is heterogeneous. Users can fake information about themselves [95] or the Web content they create [96].
Integration	Many Health Social Web applications are not integrated. However, some platforms provide open APIs to integrate third party applications [53]. Integration across different platforms can be achieved using semantic technologies [97].
Privacy-preserving extraction of personal information	Preserving privacy while user modeling and data mining [98,99]

Technological levels of maturity vary among the different approaches reviewed in this paper. Some are not only technologically feasible, but are commonly used in health personalization (eg, using PHR data to build personalized applications). Other approaches, such as the use of social network analysis to find communities of users, are technically feasible but not yet applied in health personalization. Other approaches are still experimental or too complex to be applied, such as video analysis.

The extracted information will have different levels of reliability, and whether that information can be used will depend on the application. For example, information extracted from a user profile in MySpace may be reliable enough to target a public health intervention but hardly specific enough to personalize an intervention or find subjects for a clinical trial recommender system. In addition to the reliability of the different techniques to extract information, we have to consider the validity of the sources of information. Many users tend to fake information to protect their privacy. For example, in a study of Facebook profiles, it was found that 8% of the users had fake names [95]. A similar problem is found in Web content, where tags describing content may be fake or spam [96]. The best way to ensure reliability and validity is to have human experts evaluating them. An alternative option is to rely on several data sources. In the example of the health video, it is possible to consider the keywords provided by the author and the viewers, comments, and so on. 

There are other technical challenges that are not related to the extraction of information itself, but to the different objectives of the personalization. For example, a personalized recommender system of videos for smoking cessation may suggest a video with a lung cancer x-ray. Although effective, the user may dislike and rate the video as poor. In that case, the relevance and quality of the recommendation depends on clinical parameters and not just ratings, as traditionally recommender systems do. Furthermore, different goals imply different needs of information for modeling both users and resources. A relevant parameter for a personalized application about sexual health, for example, sexual orientation, may be irrelevant in many other applications. The discussion about relevance and quality has been addressed during many years in the field of information retrieval [94,100].

In the Health Social Web, there is a wide range of data sources and applications that are not integrated. Many platforms, such as online communities, don't provide APIs for extracting information or integrating third party applications. The lack of open APIs makes it challenging to extract information for personalization and almost impossible to integrate personalized applications. However, the use of APIs is increasing as exemplified by certain PHRs that can integrate third party applications [53-55]. However, each PHR often comes with a different API, making it hard to integrate applications across different platforms. An approach to address this problem is the creation of APIs that can be used across different platforms. This approach has been applied to integrate data from different social networks platforms [97].

As explained in following subsection, one of the most important ethical challenges is how to preserve privacy while extracting information about users. That concern has motivated the creation of different data mining techniques that preserve the privacy of the “data-mined” users [98,99]. Furthermore, many Web platforms allow the users to define their own privacy preferences.

The Social Web has changed how health information and applications are being disseminated (eg, viral dissemination and collaborative filtering). Users are now relying less on traditional experts and more on guidance from fellow users within their social networks. This phenomenon, which has been termed “apomedation” [101], is already affecting personalized health applications. For example, an increasing number of applications are relying on users to be disseminated throughout their social networks [89]. This approach has implications in the evaluation of these viral applications since it may be impossible to control who uses them. One possible solution for that problem is to extract information about impact of these applications from the social network itself [93,102].

### Socioethical Challenges

While we consider ways to use available personal information to make Web content and applications more useful, we must be mindful of related ethical challenges in doing so. First and foremost among them is privacy. There is a continuum of personal information that is captured, logged, left, and made available in the Social Web. Personal health records, for example, are by definition likely to contain highly sensitive personal information and, as such, the majority of PHR providers have varied privacy and confidentiality policies as part of their terms of use. Third party applications that make use of PHR content will need to conform to stated privacy policies. However, this will not be easy as there are no standards for PHR privacy policies. As such, it will be difficult to create a single application that could be of use across different PHRs.

Existing on the other end of the continuum are those who are intentionally disclosing personal information about themselves or loved ones within blogs (eg, blogging about family genetic risks and the health of their children) [103,104]. In these contexts, privacy and confidentiality policies rarely exist, as individuals are simply free to publicly write about whatever is on their minds. When using techniques that extract user information, it is important to maintain a proper balance between the public and private nature of the content. Researchers should be mindful about common research principles, such as informed consent for using extracted information, and may consider poststudy interventions such as those used by Moreno et al [105]. Such principles can be seen in applications that first ask users if it is appropriate to use identifiable information, such as the ability to use current location to receive “geo-located” relevant content. As Wang and Kobsa suggested, there is a need to tailor privacy to the constraints of each individual user [106]. Mayer-Schonberger, on the other hand, has argued for the important historical role “forgetting” has played in society. He extends this idea to the Web in the form of expiration dates for information [107]. This deceptively simple idea would allow the erasure of certain kinds of information from the ubiquitous and eternal memory of the Web.

Another ethical issue regarding privacy is the extracting of information about minors because they are especially vulnerable to misuses of personal information. Unfortunately, disclosure of personal health information in social networks is rather common among teenagers [31]. There are different approaches to reducing it. For example, some researchers have approached minors disclosing health information on MySpace suggesting they reduce their disclosure of sensitive information by sending them emails to their profiles [108]. These messages sent to the teenagers reduced the disclosure of personal health information, but such emails may have been seen by some teenagers as spam. To avoid the risk of being seen as spammers, one possible approach is to rely on users to disseminate the intervention through their friends.

Many personalized applications within the Social Web intend to enhance socializing and sharing of knowledge between users. Unfortunately, in the health domain, there are some scenarios where the desired goal may be the opposite, since there are online communities promoting unhealthy behaviors, such as communities promoting anorexia and bulimia as “lifestyles” [109-111]. Facilitating the sharing of “proanorexic” knowledge and socializing can be harmful. However, the approaches presented in this paper can be used to identify these communities to reduce their impact (eg, parental software filtering proanorexia communities).

The integration between different data sources in the Web is partially a technical issue, but to achieve complete interoperability, there are also other barriers to be addressed. The terms of use of many Web services and APIs are complex to understand for both users and developers. In addition, these terms are normally framed within regional or national legislation, and many users may reside in locations with different legislation. For example, consumers of a company providing online direct-to-consumer genetic services, such as 23andMe, may receive online genetic counseling, which is illegal or not regulated in many countries. In addition, the laws enforcing privacy are different in each country and this affects the development of personalized applications [112]. What can be legally extracted and stored about users changes across the different countries; thus, a personalized health application may be doing something illegal while extracting information about their users depending on their residence.

### Conclusions

The Web has largely become a social platform where millions of health consumers are accessing and sharing knowledge about health [1,4]. Health consumers are not just socializing and accessing information on the Web, but are also using an increasing number of Web applications (eg, search engines and PHRs) to improve their perceived understanding of health issues. Many of these Web health applications are personalized to each user. One key aspect of health personalization in the Social Web is to extract information about users and resources. As reviewed in this paper, the Social Web offers many possibilities for the extraction of information about users and resources. It can be as simple as extracting information about age or as complex as extracting information about emotions. These techniques can be used not only for creating personalized applications but also for public health (eg, health surveillance) as part of the emerging discipline of “infodemiology” [113].

The adaptation of online intervention methodologies [114] to the context of personalization and the Social Web is an area for further research and beyond the scope of this paper. Critical issues need further exploration such as the scope and boundaries of effective online interventions, the role of trust in online health social networks and communities, and the ethical implications of research with publicly disclosed personal health information. The development of the techniques reviewed in this paper leads to new research questions: How to use the extracted information to influence health behavior in online contexts? How can we move techniques beyond individuals to groups, communities, and populations? In addition, more research is needed to determine the intrusiveness of these techniques. We need to be mindful of the issues raised in this paper, but the challenges cannot be an excuse not to develop more dynamic and personalized health applications. Outside the health domain, Web applications are becoming increasingly personalized; thus, health consumers will expect a more personalized experience in Web health applications.

The use of different approaches reviewed in this paper can catalyze the emergence of new applications adapted to the specific needs of the users without posing the traditional burden of filling in questionnaires and forms. However, in Web personalization “one size does not fit all,” so in order to decide which techniques are suitable for a specific application, we have to bear in mind the goals of the application and the personal preferences of users.

## Abbreviations

API: application programming interfaces
CCR: continuity of care record
CDA: clinical document architecture
EMR: electronic medical records
HL7: Health Level 7
NLP: natural language processing
PHR: personal health records
SNA: social network analysis
UMLS: Unified Medical Language System
